# Comprehensive evaluation of the influence of sex differences on composite disease activity indices for rheumatoid arthritis: results from a nationwide observational cohort study

**DOI:** 10.1186/s41927-023-00328-9

**Published:** 2023-03-21

**Authors:** Takahiro Nishino, Atsushi Hashimoto, Shigeto Tohma, Toshihiro Matsui

**Affiliations:** 1 Department of Rheumatology Research, Clinical Research Center for Allergy and Rheumatology, National Hospital Organization Sagamihara National Hospital, 18-1, Sakuradai, Minami-ku, Sagamihara, Kanagawa 252-0392 Japan; 2 Department of Rheumatology, National Hospital Organization Sagamihara National Hospital, Sagamihara, Kanagawa Japan; 3 Department of Rheumatology, National Hospital Organization Tokyo National Hospital, Kiyose, Tokyo Japan

**Keywords:** Rheumatoid arthritis, Disease activity, Sex difference, DAS28, SDAI, CDAI, ESR, CRP

## Abstract

**Background:**

The effects and their magnitudes of sex on disease activity indices for rheumatoid arthritis are not clear. We aimed to comprehensively evaluate the influence of sex on disease activity indices in the real-world setting using a large observational database.

**Methods:**

We analyzed 14,958 patients registered in the National Database of Rheumatic Diseases in Japan (NinJa) in 2017. We evaluated the sex differences in the 28-joint disease activity score (DAS28) using erythrocyte sedimentation rate (ESR), DAS28 using C-reactive protein (DAS28-CRP), Simplified Disease Activity Index (SDAI), and Clinical Disease Activity Index by disease activity category using Cliff’s delta and regression analysis. Differences in the share of components of indices were evaluated using permutational multivariate analysis of variance. Correction equations were constructed to estimate the number of misclassification in male patients who achieve DAS28-ESR remission.

**Results:**

DAS28-ESR showed higher values in female patients than male patients in remission despite no obvious difference in other indices or disease activity categories. Among the components of DAS28-ESR, only ESR was higher in female patients than male patients in remission. In DAS28-CRP and SDAI, 28-tender joint count was higher and CRP was lower in female patients than male patients. In addition, the profiles in the components were different between female and male patients, especially among those with high disease activity. Using correction equations, almost 12% of male patients with DAS28-ESR remission were estimated to be misclassified, mainly due to differences in ESR.

**Conclusion:**

Among the disease activity indices, significant sex difference was observed only in DAS28-ESR remission. The degree of misclassification in DAS28-ESR remission would be unignorable.

**Supplementary Information:**

The online version contains supplementary material available at 10.1186/s41927-023-00328-9.

## Introduction

The concept of “Treat to Target” (T2T), which involves setting a goal and determining an appropriate treatment, has improved outcomes in patients with rheumatoid arthritis (RA) [1–3]. Assessing the disease activity is an important part of T2T. Disease activity is usually evaluated using scoring systems, such as 28-joint disease activity score (DAS28) using erythrocyte sedimentation rate (ESR), DAS28 using C-reactive protein (DAS28-CRP), Clinical Disease Activity Index (CDAI), and Simplified Disease Activity Index (SDAI) [4–7]. Although these indices are widely used and recommended to assess RA disease activity [8], they are influenced by sex, age, body mass index, and other factors [9–21]. These effects and their magnitude on disease activity indices are not clear; therefore, disease activity should be carefully interpreted based on the properties of each index and individual patient factors.

Sex differences in RA have been studied from multifaceted aspects, for example, incidence, phenotype, comorbidities, treatment response, and prognosis [22]. In addition, sex differences in disease activity indices have been studied, mainly in DAS28-ESR. Many studies have reported that DAS28-ESR is lower in male patients compared to female patients [10–16], and ESR is considered to contribute to the sex differences [10, 12, 13]. The association of sex difference with CRP level and discrepancy between DAS28-ESR and DAS28-CRP have been studied [16, 23–27], but few studies have evaluated the effect of sex differences on DAS28-CRP [16]. Although the effects of sex differences on CDAI and SDAI have not been thoroughly evaluated, these indices are influenced by pain perception and sex [9]. Therefore, the effect of sex differences on these indices cannot be ignored. Nevertheless, sex differences are not taken into account while assessing disease activity and it is not clear how sex differences influence the composite measure indices, which may lead to biased interpretation of these indices. The current problem is a lack of large-scale systematic evaluation of the effects of sex differences on disease activity indices in the era of biologics and molecular targeting therapy. Most previous studies that evaluated the effects of sex differences on disease activity indices were conducted in the 2000s and involved less than 1000 patients. Since then, the profile of drugs used in RA patients has changed. However, the effects of sex differences on disease activity indices are not well clear in patients treated with biologics or molecular targeting therapy. Furthermore, most previous studies only performed a simple comparison of the disease activity indices without stratification, which may introduce bias in the results due to the combined analysis of patients with varying disease activity. Thus, it is unclear whether the previous studies accurately evaluated the effects of sex differences on disease activity indices or the results were obtained due to differences in disease activity between the groups. For the aforementioned reasons, the effects of sex differences on disease activity indices, while taking into account the different drugs used, require a comprehensive evaluation. Therefore, we aimed to evaluate the influence of sex differences on composite measure indices and their clinical impact by analyzing the impact on each disease activity category using a large nationwide observational database. The results would enhance our understanding and allow more appropriate use of composite measure indices by taking into account the sex differences.

## Patients and methods

### Study population

We collected data from a nationwide observational cohort database of RA in Japan (National Database of Rheumatic Diseases in Japan; NinJa) [26, 28] in 2017. Forty-nine hospitals and institutions from all over Japan participated in the NinJa project in 2017. NinJa included RA patients diagnosed according to the standard diagnostic criteria for RA [29–32], regardless of disease duration, onset age, and treatment. Once a year, NinJa collects information about important events (e.g., hospitalization, surgical operation, malignancy, tuberculosis, herpes zoster, or childbirth) and data are arbitrarily collected at one point in the year for each patient, including 28-tender joint count (TJC28), 28-swollen joint count (SJC28), disease activity indices, Health Assessment Questionnaire-Disability Index (HAQ-DI), drug use, and joint destruction. The collected data are curated in National Hospital Organization Sagamihara National Hospital and verified in case of doubt about accuracy. Of a total of 15,185 patients registered in NinJa in 2017, 15,056 had onset of RA at age > 16 years. We analyzed 14,958 out of 15,056 patients, thereby excluding 98 patients in whom the drug used was unknown (94 patients) or not approved for RA in Japan (4 patients).

### Measures and disease activity categories

We evaluated the influence of sex differences on DAS28-ESR, DAS28-CRP, CDAI, and SDAI. Patients were classified into remission, low disease activity (LDA), moderate disease activity (MDA), and high disease activity (HDA) based on DAS28-ESR, DAS28-CRP, CDAI, and SDAI, in accordance with the updated American College of Rheumatology recommendations [8].

### Statistical analysis

Statistical analyses were performed using R version 4.0.3 software (R Foundation for Statistical Computing). The URLs and/or references of R packages and function are listed in the Additional file 1. The values and graphs are the results of available-case analysis (pairwise deletion), unless stated otherwise. Figures were generated using *ggplot2* or *car* package or geom_flat_violin function. To compare continuous variables, differences in 25% trimmed mean between female and male patients were calculated and its 95% confidence intervals (CIs) were obtained by the percentile bootstrap method (5000 iterations) using *simpleboot* and *boot* packages. Cliff’s delta, non-parametric effect size, and its 95% CIs were calculated using *effsize* package for comparing disease activity indices and their components between female and male patients. The magnitude of effect size based on Cliff’s delta for disease activity indices are not established, thus thresholds of magnitude for the absolute values were assessed that less than 0.147, 0.147 or more and less than 0.330, 0.330 or more and less than 0.474, and 0.474 or more corresponded to negligible, small, medium, and large according to the threshold values proposed by Romano et al. [33].

Permutational multivariate analysis of variance (PERMANOVA) was performed using *vegan* package for examining the difference in the share of components to disease activity indices between female and male patients in each disease activity category (number of permutations: 1000). The share of components is considered to be compositional data containing essential zeros, which cannot calculate Aitchison’s distance. Thus, Bray–Curtis dissimilarity was applied for the analysis of PERMANOVA. The threshold of p-value was not defined because this study used observational database without prespecified analysis plan and sample size design to control for type I error.

The results of regression analysis were confirmed by generalized linear model (GLM) with gamma distribution using identity link and quantile (median) regression (QR) model. Although the canonical link function of GLM with gamma distribution provides a reciprocal link, we used identity link because coefficients can be interpreted in terms of the effects of independent variables on disease activity indices at the original scale. Dependent variable in GLM with gamma distribution should not contain zero or negative values. In case that the remission dataset to be analyzed in GLM contained patients whose disease activity indices were zero, a small value (1.0 × 10^–15^) was added to the value for all patients in the dataset. QR was performed using *quantreg* package.

In regression models adjusting for patient-related factors, analysis using stacked dataset imputed by chained equations were also performed in addition to the available-case analysis to confirm the robustness of the results. Multivariate imputation by chained equations were conducted using *mice* package. Imputation method and models are described in the Supplementary Methods (see Additional file 1). As conventional procedure of multiple imputation, the coefficients of regression models were pooled by Rubin’s rule. However, imbalance of cases between imputed datasets occurs by stratifying disease activity categories due to imputation of the disease activity indices; therefore, the results cannot be combined by Rubin’s rule. Alternatively, we analyzed the stacked imputed dataset, which was deemed as the complete data. The point estimates calculated by the stacked method are unbiased, but the confidence intervals are invalid [34]. Thus, we present the coefficients and their confidence intervals in available-case analysis as the main results.

## Results

### Patient characteristics

Among a total of 14,958 patients analyzed in this study, 11,916 were female patients (79.7%) and 3042 were male patients (20.3%). Table 1 presents the patient characteristics. Male patients were older and had a higher age at onset, whereas female patients had longer disease duration, higher HAQ-DI score, and higher values of disease activity indices. Male patients had higher remission rate than female patients in all disease activity indices, especially DAS28-ESR. Of the 14,958 patients, 1043 were not treated with disease modifying anti-rheumatic drugs (DMARDs), 9593 were treated with conventional synthetic DMARDs (csDMARDs) alone, 2100 were treated with tumor necrotizing factor inhibitors (TNFi), 1144 were treated with interleukin 6 receptor inhibitors (IL-6i), 772 were treated with cytotoxic T lymphocyte-associated antigen 4 immunoglobulin (CTLA-4-Ig), and 306 were treated with Janus kinase inhibitors (JAKi). Additional file 1: Tables S1–S6 present the patient characteristics by sex for each treatment type (see Additional file 1).

**Table 1:** Patient characteristics

	Female (n = 11,916)	Male (n = 3042)	Δ25% trimmed mean (95% CI)
Age, years	68 (58–75)	69 (62–76)	− 1.8 (− 2.3 to − 1.3)
Age at onset, years	52 (41–62)	59 (50–67)	− 6.9 (− 7.5 to − 6.3)
Disease duration, years	11 (6–20)	8 (4–14)	3.6 (3.2 to 3.9)
Number of artificial joints	0 (Q1–Q3, 0–0; range, 0–9)	0 (Q1–Q3, 0–0; range, 0–7)	0.0 (0.0 to 0.0)
Stage			
I	2752 (25.3)	980 (35.6)	–
II	2908 (26.8)	928 (33.7)	–
III	2094 (19.3)	479 (17.4)	–
IV	3103 (28.6)	363 (13.2)	–
Missing data	1059	292	–
Class			
I	3762 (34.6)	1247 (45.0)	–
II	4960 (45.6)	1168 (42.2)	–
III	1848 (17.0)	306 (11.0)	–
IV	314 (2.9)	50 (1.8)	–
Missing data	1032	271	–
BMI, kg/m^2^	21.92 (3.73)	23.01 (3.38)	− 1.29 (− 1.44 to − 1.15)
Missing data	1722	422	
Steroid			
Regular use	4110 (34.5)	1137 (37.4)	–
Missing data	0	1	–
NSAIDs			
Regular use	3609 (30.3)	959 (31.5)	–
Missing data	1	0	–
RF, IU/mL	44.0 (14.0–121.0)	45.0 (10.0–160.2)	− 5.69 (− 11.27 to − 0.67)
Positive (> 15)	6872 (73.7)	1614 (68.6)	–
Missing data	2598	690	–
Anti-CCP, U/mL	52.9 (3.6–266.0)	60.5 (0.7–338.5)	− 16.00 (− 34.00 to 0.72)
Positive (≥ 4.5)	2970 (74.1)	806 (67.7)	–
Missing data	7908	1851	–
HAQ-DI	0.38 (0.00–1.00)	0.00 (0.00–0.50)	0.275 (0.249 to 0.301)
Missing data	2991	777	–
Smoking status			
Never	7734 (79.1)	625 (24.7)	–
Former	1386 (14.2)	1322 (52.3)	–
Current	654 (6.7)	583 (23.0)	–
Missing data	2142	512	–
DAS28-ESR	2.9 (2.2–3.7)	2.5 (1.8–3.3)	0.39 (0.33 to 0.45)
Remission	3552 (40.7)	1183 (53.9)	–
Low	1857 (21.3)	386 (17.6)	–
Moderate	2891 (33.1)	531 (24.2)	–
High	428 (4.9)	94 (4.3)	–
Missing data	3188	848	–
DAS28-CRP	2.1 (1.5–2.9)	2.0 (1.5–2.8)	0.09 (0.04 to 0.13)
Remission	6868 (67.1)	1815 (69.5)	–
Low	1509 (14.7)	362 (13.9)	–
Moderate	1691 (16.5)	387 (14.8)	–
High	170 (1.7)	46 (1.8)	–
Missing data	1678	432	–
CDAI	4.8 (1.9–9.2)	3.7 (1.3–7.8)	1.05 (0.82 to 1.29)
Remission	3537 (34.7)	1110 (42.6)	–
Low	4483 (43.9)	1060 (40.7)	–
Moderate	1851 (18.1)	363 (13.9)	–
High	333 (3.3)	72 (2.8)	–
Missing data	1712	437	–
SDAI	5.2 (2.1–9.9)	4.2 (1.6–8.6)	0.88 (0.62 to 1.12)
Remission	3684 (36.2)	1101 (42.3)	–
Low	4388 (43.1)	1053 (40.5)	–
Moderate	1853 (18.2)	382 (14.7)	–
High	247 (2.4)	64 (2.5)	–
Missing data	1744	442	–

The values are n, n (%), mean (SD) or median (Q1–Q3). Number of artificial joints represents median, Q1–Q3 and range. *SD* Standard deviation; *Q1* First quartile; *Q3* Third quartile; *95% CI* 95% confidence interval

If categorical data contains missing data, the percentages are calculated with its denominator as the number subtracting the number of missing data from total number. If continuous data contains missing data, the representative values are the results of available-case analysis

The total of percentage may not equal to 100 due to rounding

The value of difference in 25% trimmed mean (Δ25% trimmed mean) is calculated by subtracting the male value from the female value

### Sex differences in DAS28-ESR, DAS28-CRP, CDAI, and SDAI by disease activity category

**Fig. 1:**
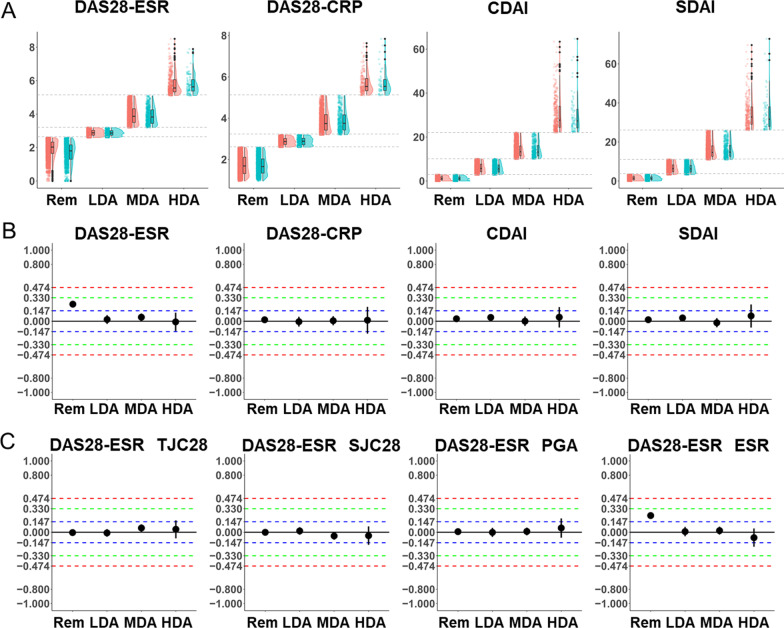
Comparison of DAS28-ESR, DAS28-CRP, CDAI, and SDAI between female and male patients. **A** Distributions of disease activity indices by sex. Jitter, box, and violin plots are depicted in each disease activity category by sex (red, female; blue, male). Dashed lines are drawn on the cutoff values for the disease activity categories (DAS28-ESR and DAS28-CRP: 2.6, 3.2, and 5.1. CDAI: 2.8, 10, and 22. SDAI: 3.3, 11, and 26). **B**, **C** Cliff’s delta for sex difference in disease activity indices. Positive values of Cliff’s delta indicate that the values of the indices and their components were higher in female patients compared to male patients, whereas negative values indicate the opposite. Black line is drawn at the value of 0.000. Blue, green, and red dashed lines are drawn on the values of 0.147, 0.330, and 0.474, respectively. Points and bars indicate the estimates and 95% CIs of Cliff’s delta, respectively. Rem, remission; LDA, low disease activity; MDA, moderate disease activity; HDA, high disease activity

A comparison of the distributions of DAS28-ESR, DAS28-CRP, CDAI, and SDAI values between female and male patients by disease activity category showed no difference, except for remission in DAS28-ESR (Fig. 1A). Cliff’s delta also showed sex difference only in DAS28-ESR remission, with higher values in female patients than male patients (Fig. 1B). Furthermore, we evaluated the sex difference in DAS28-ESR components and explored whether the components of these indices that showed no sex differences were less sensitive to sex difference. Cliff’s delta showed that ESR was higher in female patients than male patients in remission, while other components of DAS28-ESR and ESR in other disease activity categories showed no obvious sex differences (Cliff’s delta for each component of DAS28-ESR are shown in Fig. 1C and that of other indices are shown in Additional file 1: Fig. S1; the values of Cliff’s delta are shown in Additional file 1: Table S7). Therefore, we concluded that sex difference in DAS28-ESR remission was mainly caused by sex difference in ESR. Cliff’s delta was also calculated for each treatment group (Additional file 1: Table S7), which showed higher DAS28-ESR values and ESR for female patients in the DMARDs free, csDMARDs, TNFi, and IL-6i groups compared to male patients in remission; however, the results for CTLA-4-Ig and JAKi were equivocal.

Sex differences were also observed in TJC28 and CRP in DAS28-CRP and SDAI (Additional file 1: Fig. S1, Table S7). In DAS28-CRP and SDAI, TJC28 tended to be higher in female patients compared to male patients in LDA to MDA and showed obvious difference in HDA. In contrast, CRP tended to be higher in male patients compared to female patients in remission to MDA and showed obvious difference in HDA in DAS28-CRP and SDAI. No obvious differences were observed in the other components of DAS28-CRP and SDAI. In each treatment group, similar results were observed in DMARDs free and csDMARDs groups (Additional file 1: Table S7). However, in the other treatment groups, the sex differences in TJC28 and CRP were equivocal or could not be evaluated due to small sample size.

### Profile in the share of components of disease activity indices

**Fig. 2:**
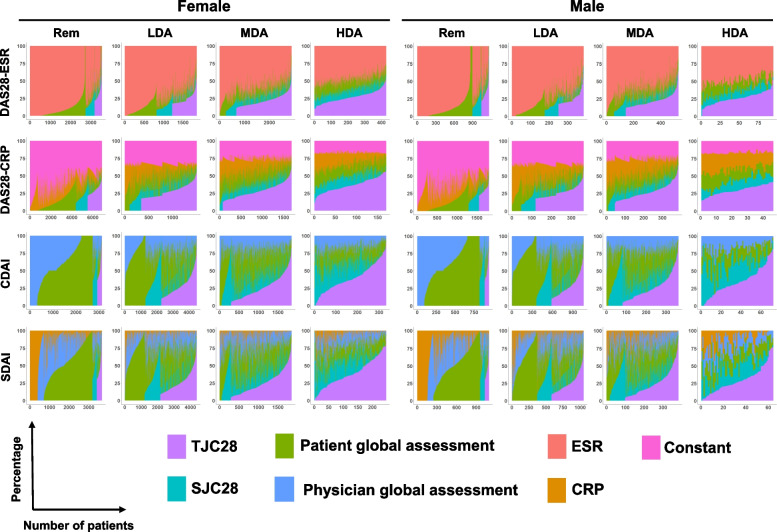
Share of the components of disease activity indices by sex. The stacked bar plot shows the share in each patient. The horizontal axis indicates the number of patients and the vertical axis indicates percentage of share of each component. Rem, remission; LDA, low disease activity; MDA, moderate disease activity; HDA, high disease activity

Considering the contrasting dynamics of TJC28 and CRP as well as no obvious differences in the other components of DAS28-CRP and SDAI, DAS28-CRP and SDAI had no sex differences because TJC28 and CRP cancel each other. Therefore, the share of each component of the composite measures would be different between female and male patients, even if the values of the composite measures are the same. We compared the share of components between female and male patients. The share of each component to the total value of the composite measures was calculated using the procedure described by Radovits et al. [10], after excluding patients whose disease activity index value was zero. The share of components of each composite measure by disease activity category is presented in Fig. 2 and was similar between female and male patients, except for HDA in DAS28-CRP and SDAI. In HDA, the share of TJC28 in DAS28-CRP and SDAI was slightly lower in male patients compared to female patients. Distributions of the share supported that the share of TJC28 tended to be higher in female patients compared to male patients and that of CRP was the opposite in the two indices (Additional file 1: Fig. S2). Furthermore, we performed PERMANOVA to quantify the impact of sex on the share (Additional file 1: Table S8). In HDA, the R^2^ values in DAS28-CRP and SDAI were relatively higher than others, while sex only partly explained the variance (DAS28-CRP, R^2^ = 0.06577; SDAI, R^2^ = 0.02520). Thus, we concluded that the composition was similar between female and male patients, but the component profile in HDA slightly differed in DAS28-CRP and SDAI.

### Adjustment of patient-related factors by regression models

We performed multivariable regression analysis by disease activity category to adjust patient-related factors using GLM and QR model. In both GLM and QR model, sex differences were only seen in remission in DAS28-ESR after adjustment of patient-related factors (Table 2 shows only the results of “Male” variable; the results of all variables are shown in Additional file 1: Table S9). The regression analysis using stacked dataset imputed by chained equations showed similar results (Additional file 1: Table S10).

**Table 2:** Partial regression coefficients of sex in GLM and QR models for adjustment of patient-related factors

	Remission	LDA	MDA	HDA
	Estimate (95% CI)	Estimate (95% CI)	Estimate (95% CI)	Estimate (95% CI)
DAS28-ESR (GLM)	− 0.281 (− 0.318 to − 0.244)	− 0.003 (− 0.024 to 0.017)	− 0.029 (− 0.079 to 0.023)	0.011 (− 0.131 to 0.156)
DAS28-ESR (QR)	− 0.235 (− 0.285 to − 0.187)	0.000 (− 0.022 to 0.025)	− 0.033 (− 0.092 to 0.066)	0.016 (− 0.131 to 0.149)
DAS28-CRP (GLM)	0.026 (0.002 to 0.051)	0.004 (− 0.019 to 0.027)	0.039 (− 0.022 to 0.100)	0.136 (− 0.066 to 0.342)
DAS28-CRP (QR)	0.053 (0.010 to 0.094)	0.008 (− 0.031 to 0.051)	0.036 (− 0.040 to 0.142)	0.036 (− 0.168 to 0.255)
CDAI (GLM)	− 0.017 (− 0.078 to 0.046)	− 0.072 (− 0.219 to 0.079)	0.126 (− 0.277 to 0.538)	0.314 (− 1.941 to 2.681)
CDAI (QR)	0.033 (− 0.081 to 0.091)	− 0.032 (− 0.231 to 0.221)	0.140 (− 0.484 to 0.743)	− 0.484 (− 2.355 to 1.840)
SDAI (GLM)	0.013 (− 0.055 to 0.084)	− 0.058 (− 0.215 to 0.102)	0.250 (− 0.231 to 0.743)	0.821 (− 1.629 to 3.374)
SDAI (QR)	0.054 (− 0.042 to 0.129)	0.013 (− 0.204 to 0.219)	0.563 (− 0.007 to 1.247)	0.184 (− 2.059 to 2.523)

*GLM* Generalized linear model; *QR* Quantile regression; *LDA* Low disease activity; *MDA* Moderate disease activity; *HDA* High disease activity

### Estimation of the impact of sex difference on remission rate

To quantify the clinical impact of sex difference in DAS28-ESR remission, we constructed correction equations using GLM and QR model. Previous studies suggest that ESR levels change in a sex specific age-dependent manner [10, 35]. Assuming that tender and swollen joint count is surrogate index which accurately reflects disease activity under the condition ESR and DAS28-ESR may not accurately reflect disease activity, we used regression model with DAS28-ESR as a dependent variable and age, 0.56 × √(TJC28) + 0.28 × √(SJC28), sex, interaction term of sex and age, and interaction term of sex and 0.56 × √(TJC28) + 0.28 × √(SJC28) as independent variables for patients with DAS28-ESR remission. We calculated the coefficients using both GLM and QR model (Table 3). Using the results of regression analysis, we constructed the following correction equations (Eqs. 1 and 2).

1 $$0.825 - 0.008 \times {\text{age}} - 0.177 \times \left( {0.56 \times \surd \left( {{\text{TJC}}28} \right) + 0.28 \times \surd \left( {{\text{SJC}}28} \right)} \right)$$

2 $$1.021 - 0.011 \times {\text{age}} - 0.202 \times \left( {0.56 \times \surd \left( {{\text{TJC}}28} \right) + 0.28 \times \surd \left( {{\text{SJC}}28} \right)} \right)$$

**Table 3:** Regression analysis for construction of correction equations

	GLM	QR
	Estimate (95% CI)	Estimate (95% CI)
Intercept	1.407 (1.325 to 1.490)	1.503 (1.375 to 1.614)
Age	0.008 (0.006 to 0.009)	0.007 (0.006 to 0.010)
Joint	0.426 (0.351 to 0.503)	0.384 (0.333 to 0.449)
Male	− 0.825 (− 0.983 to − 0.664)	− 1.021 (− 1.229 to − 0.686)
Age: male	0.008 (0.006 to 0.010)	0.011 (0.006 to 0.014)
Joint: male	0.177 (0.040 to 0.318)	0.202 (0.061 to 0.297)

The variable “Joint” represents 0.56 × √(TJC28) + 0.28 × √(SJC28)

The variable “Male” is dummy variable that female is used as reference

The variable “Age: Male” represents interaction term of age and male sex

The variable “Joint: Male” represents interaction term of joint findings and male sex

*GLM* Generalized linear model; *QR* Quantile regression

**Fig. 3:**
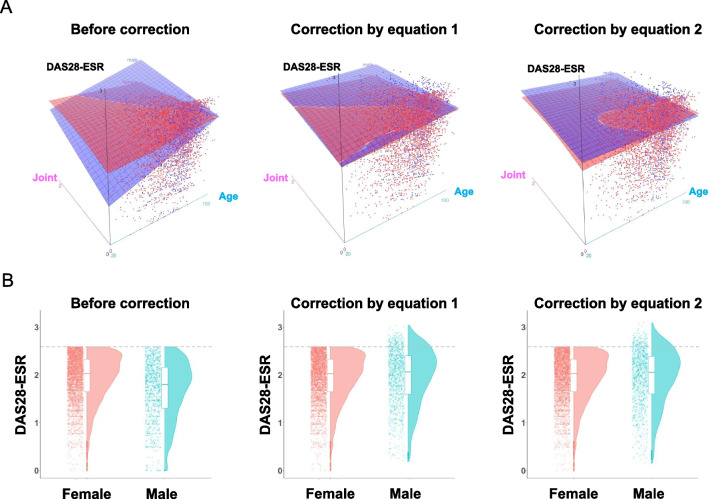
The effect of correction by equations on DAS28-ESR. **A** Relationship between DAS28-ESR, age, and joint findings before and after corrections. Three-dimensional scatter plot with fitted surface are depicted by sex (red, female; blue, male). “Joint” represents 0.56 × √(TJC28) + 0.28 × √(SJC28). **B** Distributions of DAS28-ESR before and after corrections. Jitter, box, and violin plots are depicted for each disease activity category by sex (red, female; blue, male). Dashed lines are drawn at the value of 2.6 in DAS28-ESR

If the values of equations are above zero, they were added to DAS28-ESR. If the values are zero or below, the original DAS28-ESR value was used. These equations were only applied to male patients. After correction using the above equations, sex difference was deemed to be negligible (Fig. 3A and Additional file 1: Fig. S3). Cliff’s delta indicated negligible sex difference after correction [Eq. (1), − 0.0530 (95% CI − 0.0887 to − 0.0172); Eq. (2), − 0.0446 (95% CI − 0.0800 to − 0.0090)]. Of the total 1183 male patients in DAS28-ESR remission (available-case analysis), 143 (almost 12.1%) and 141 (almost 11.9%) male patients in DAS28-ESR remission were classified as LDA by correction using Eqs. 1 and 2, respectively (Fig. 3B). Therefore, almost 12% of male patients who achieve DAS28-ESR remission criteria are estimated to be misclassified.

## Discussion

This study confirmed that among DAS28-ESR, DAS28-CRP, CDAI, and SDAI, sex difference in the composite measures is observed only in remission based on DAS28-ESR. In addition, sex difference in DAS28-ESR remission is mainly due to difference in ESR, which is consistent with previous studies [12, 13, 36]. However, our results showed no meaningful sex differences in other components of DAS28-ESR, which is not consistent with previous studies [12–14, 36]. In previous studies, tender joint count and patient global assessment values, as well as ESR, were consistently higher in female patients compared to male patients. The reason for this discrepancy is uncertain, but may result from differences in stratification and study population (e.g., remission rate or pain intensity). The previous studies compared female and male patients without stratification or with stratification by swollen joint count, while we stratified patients by disease activity based on each composite measure. Therefore, the populations analyzed in the previous studies may have included patients with various disease activities than our analysis. Both tender joint count and patient global assessment are affected by pain [13, 37]. Although the mechanisms underlying sex difference in pain perception are not fully clear, female sex is more sensitive to pain than male sex [38, 39]. Thus, pain may contribute to sex differences in composite measures. However, adjusting for individual differences in pain intensity is challenging because the most frequently used measures, Visual Analog Scale and Numeric Rating Scale, are subjective and dependent on individual pain tolerance and other factors [40].

We found that the values of composite measures do not differ between female and male patients, but profile in the share of components differ between the sexes in composite measures including CRP. Several studies reported that sex difference is not observed in CRP level in RA patients [13, 36], and it is widely believed that CRP is less sensitive to sex difference than ESR. However, some studies reported that the CRP level is higher in male patients compared to female patients, similar to the results of our study [14, 16, 23]. Although, this discrepancy needs to be evaluated in further studies, our findings suggest that results of clinical trials for patients whose disease activity is assessed as HDA by DAS28-CRP and SDAI may be carefully interpreted because of the possibility that study population is not homogeneous between female and male patients. Compared to DAS28-ESR, sex differences in DAS28-CRP, CDAI, and SDAI have not been well studied. This study revealed behaviors of DAS28-CRP, CDAI, and SDAI in sex difference using stratification by disease activity category and evaluation by effect size at the level of components. The importance of our results is that they clarify whether or not disease activity indices are affected by sex differences in each disease activity category. Evidence for robustness to sex differences is necessary for the appropriate selection of indices to be used in clinical trials and routine clinical practice. Based on the findings of our study, CDAI is the most robust to sex difference among the four indices. Our study provides a cautionary implication that composite measure indices should be carefully interpreted in light of confounders, such as sex difference, before making clinical decisions based on the values of composite measure indices.

In this study, we also found that almost 12% of male patients may be misclassified as being in remission based on DAS28-ESR, suggesting that the criterion overestimates the remission rate in male patients. Generally, remission rate is lower in female patients compared to male patients, and male sex is a predictor of remission [41, 42]. In our study, sex difference had a greater effect on remission rate defined using the DAS28-ESR criteria compared to other criteria; however, to a lesser extent, the remission rates were higher in all disease activity indices in male patients compared to female patients. Previous studies showed that the remission rate was lower in female patients compared to male patients using DAS28-ESR, but not when other criteria were used, thereby suggesting that the sex difference in remission rate based on DAS28-ESR is due to sex difference in ESR [36, 43]. Although further study is needed to determine whether sex difference in remission rate may be explained by factors other than bias caused by composite measures themselves, e.g., pathophysiological or psychosocial differences, most part of the sex difference in remission rate based on DAS28-ESR would be explained by ESR [12, 36]. Overestimation of the remission rate due to sex difference in ESR is important in the context of determining treatment outcomes. In early RA, achieving remission confer favorable radiographic, quality of life and functional outcome compared to LDA [44]. Thus, misclassification of LDA into remission would have great impact on clinical decision making in T2T era, while further studies are needed to investigate the effects of misclassification of male patients with LDA into remission due to ESR on the radiographic appearance, quality of life, and functional outcome in both early and established RA using longitudinal data.

The present study had several limitations. First, we did not analyze all the factors that affect ESR and other components of the indices, such as hematocrit level, alcohol consumption, race, ethnicity, and history of fibromyalgia, hypergammaglobulinemia, Sjögren's syndrome, or other comorbidities. Second, the correction equations were not validated in an independent dataset. Therefore, correction using these equations is not yet suitable for clinical use, and estimated misclassification rates may fluctuate especially in applying to non-Japanese populations. Moreover, the equations and estimations are based on the assumption that tender and swollen joint count accurately reflects disease activity because no unbiased gold standard measure of disease activity has been established. The definitions of disease activity and remission are complex and controversial with respect to whether they should be defined solely by the state of inflammation (e.g., joint findings, ESR, or CRP) or by the combination of patients’ overall health status (e.g., patient global assessment) and state of inflammation [45, 46]. The dual target strategy, which separately manages inflammation measures as disease activity and patient-reported outcomes as disease impact, may avoid overtreatment and improve the quality of life of patients [46, 47]. Patient-reported outcomes are not only proposed as the pillar of the dual target strategy, but are also associated with functional outcomes and sustained remission [48–50]. Thus, it is unclear whether the assumption that joint findings are a proxy for disease activity is optimal. However, to the best of our knowledge, this is the first report to quantitatively determine the impact of bias due to sex difference on remission using correction equations, and these are candidates to correct sex difference in remission based on DAS28-ESR.

## Conclusion

Large-scale and detailed analysis, including the drug type used, of the disease activity indices showed significant sex differences only in DAS28-ESR remission, mainly due to differences in ESR. In DAS28-CRP and SDAI, the values of the composite measures showed no significant sex differences, but TJC28 was higher and CRP was lower in female patients than male patients. This sex difference in the components indicated that the profiles of male and female patients were different, especially in those with high disease activity. Furthermore, almost 12% of male patients with DAS28-ESR remission were considered to be equivalent to LDA using equations to correct the effects of sex and age differences on ESR. Our results will help to understand the properties of composite measures of disease activity and allow the appropriate selection of indices based on the sex differences.

## Supplementary Information

**Additional file 1: Methods S1.** Imputation method and models for multiple imputation by chained equations. **Methods S2.** Predictor matrix. **Fig. S1.** Cliff’s delta for sex difference in each component in available-case analysis. **Fig. S2.** Distributions of the share of each component. **Fig. S3.** Relationships between DAS28-ESR and age or joint findings before and after correction. **Table S1.** Patient characteristics (DMARDs free). **Table S2.** Patient characteristics (csDMARDs). **Table S3.** Patient characteristics (TNFi). **Table S4.** Patient characteristics (IL-6i). **Table S5.** Patient characteristics (CTLA-4-Ig). **Table S6.** Patient characteristics (JAKi). **Table S7.** Cliff’s delta for sex difference in disease activity indices and their components by treatment type. **Table S8.** PERMANOVA for sex difference in the share of components. **Table S9.** Adjustment for patient-related factors with GLM and QR in available-case analysis. **Table S10.** Adjustment for patient-related factors with GLM and QR using stacked dataset imputed by chained equations.

## Data Availability

The datasets generated and/or analysed during the current study are not publicly available because they contain patient information and unrestricted access to them is not permitted by the ethics committee. But they are available from the corresponding author on reasonable request subject to ethical approval and with permission of all investigators participating this study.

## Abbreviations

CDAI: Clinical Disease Activity Index
CIs: Confidence intervals
CRP: C-reactive protein
csDMARDs: Conventional synthetic disease modifying anti-rheumatic drugs
CTLA-4-Ig: Cytotoxic T lymphocyte-associated antigen 4 immunoglobulin
DAS28: 28-joint disease activity score
DMARDs: Disease modifying anti-rheumatic drugs
ESR: Erythrocyte sedimentation rate
GLM: Generalized linear model
HAQ-DI: Health Assessment Questionnaire-Disability Index
HDA: High disease activity
IL-6i: Interleukin 6 receptor inhibitors
JAKi: Janus kinase inhibitors
LDA: Low disease activity
MDA: Moderate disease activity
NinJa: National Database of Rheumatic Diseases in Japan
PERMANOVA: Permutational multivariate analysis of variance
QR: Quantile regression
RA: Rheumatoid arthritis
SDAI: Simplified Disease Activity Index
SJC28: 28-swollen joint count
T2T: Treat to Target
TJC28: 28-tender joint count
TNFi: Tumor necrotizing factor inhibitors
