# Supplementary material for: Comprehensive evaluation of the influence of sex differences on composite disease activity indices for rheumatoid arthritis: results from a nationwide observational cohort study
Source: BMC Rheumatol. 2023 Mar 21;7:4. doi: 10.1186/s41927-023-00328-9 (PMC10029312; doi:10.1186/s41927-023-00328-9)
Supplement: Supplementary file 1 — Additional file 1: Methods S1. Imputation method and models for multiple imputation by chained equations. Methods S2. Predictor matrix. Fig. S1. Cliff’s delta for sex difference in each component in available-case analysis. Fig. S2. Distributions of the share of each component. Fig. S3. Relationships between DAS28-ESR and age or joint findings before and after correction. Table S1. Patient characteristics (DMARDs free). Table S2. Patient characteristics (csDMARDs). Table S3. Patient characteristics (TNFi). Table S4. Patient characteristics (IL-6i). Table S5. Patient characteristics (CTLA-4-Ig). Table S6. Patient characteristics (JAKi). Table S7. Cliff’s delta for sex difference in disease activity indices and their components by treatment type. Table S8. PERMANOVA for sex difference in the share of components. Table S9. Adjustment for patient-related factors with GLM and QR in available-case analysis. Table S10. Adjustment for patient-related factors with GLM and QR using stacked dataset imputed by chained equations. [file 41927_2023_328_MOESM1_ESM.docx]

**Supplementary Data**

**Comprehensive evaluation of the influence of sex differences on composite disease activity indices for rheumatoid arthritis: results from a nationwide observational cohort study**

Takahiro Nishino, Atsushi Hashimoto, Shigeto Tohma, Toshihiro Matsui

**Table of contents**

- **Supplementary Methods**

1. Imputation method and models for multiple imputation by chained equations 1
2. Predictor matrix 1-2

- **Supplementary Figures and Legends**

1. Figure S1. Cliff’s delta for sex difference in each component in available-case analysis 3
2. Figure S2. Distributions of the share of each component 4
3. Figure S3. Relationships between DAS28-ESR and age or joint findings before and after correction 5
4. Legends 6

- **Supplementary Tables and Legends**

1. Table S1. Patient characteristics (DMARDs free) 7
2. Table S2. Patient characteristics (csDMARDs) 8
3. Table S3. Patient characteristics (TNFi) 9
4. Table S4. Patient characteristics (IL-6i) 10
5. Table S5. Patient characteristics (CTLA-4-Ig) 11
6. Table S6. Patient characteristics (JAKi) 12
7. Table S7. Cliff’s delta for sex difference in disease activity indices and their components by treatment type 13-16
8. Table S8. PERMANOVA for sex difference in the share of components 17
9. Table S9. Adjustment for patient-related factors with GLM and QR in available-case analysis 18-19
10. Table S10. Adjustment for patient-related factors with GLM and QR using stacked dataset imputed by chained equations 20
11. Legends 21-22

- **URLs and/or references of R packages and function** 23

**Supplementary Methods**

1. Imputation method and models for multiple imputation by chained equations

The database contained missing values due to missing patient responses, not performing physical examination or laboratory tests, and/or no response to the questionnaire. A high proportion of missing values for anti-cyclic citrullinated peptide (anti-CCP) was assumed to be because the Japanese health insurance system limits the number of anti-CCP tests that can be performed. Imputation models were constructed using age, age at onset, disease duration, male sex, number of artificial joints, NSAIDs use, steroid use, stage, class, BMI, RF, anti-CCP, HAQ-DI, smoking status, DAS28-ESR, DAS28-CRP, CDAI, SDAI, TJC28, SJC28, patient global assessment, physician global assessment, ESR, CRP, joint findings (0.56 × √(TJC28) + 0.28 × √(SJC28)), interaction term of age and male sex, and interaction term of joint findings and male sex as variables. We applied predictive mean matching to continuous variables and logistic regression or polytomous logistic regression model to categorical variables. Combinations of target variables to be imputed and variables used to impute target variables are listed in the Predictor matrix. Variables in the rows are target variables and variables in the columns are variables used to impute the target variables. The number of multiple imputations was set to 100. Thus, the total sample size of analysis using stacked dataset imputed by chained equations is 1,495,800 (number of multiple imputations [100] × sample size [14,958] = 1,495,800).

1. Predictor matrix

Legend:

“1” in the matrix indicates that the variable in the column is used to impute the target variable in the row, and “0” indicates it is not used as variable to impute the target variable in the row.

“Joint” represents 0.56 × √(TJC28) + 0.28 × √(SJC28).

The variable “Age : Male” represents interaction term of age and male sex.

The variable “Joint : Male” represents interaction term of joint findings and male sex.

TJC28, 28-tender joint count; SJC28, 28-swollen joint count; PGA, patient global assessment; PhGA, physician global assessment.

Predictor matrix

**Supplementary Figure 1.** Cliff’s delta for sex difference in each component in available-case analysis


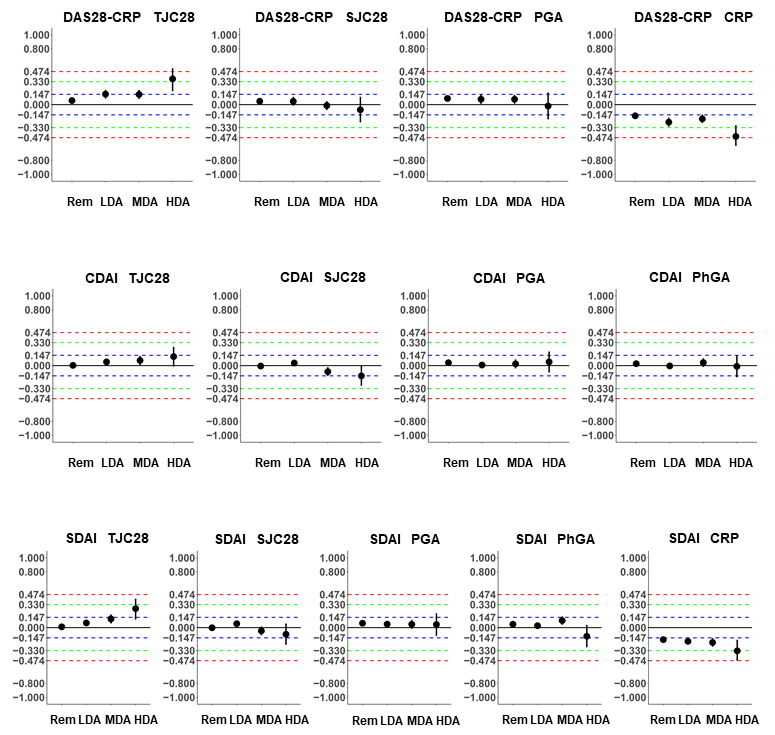


**Supplementary Figure 2.** Distributions of the share of each component


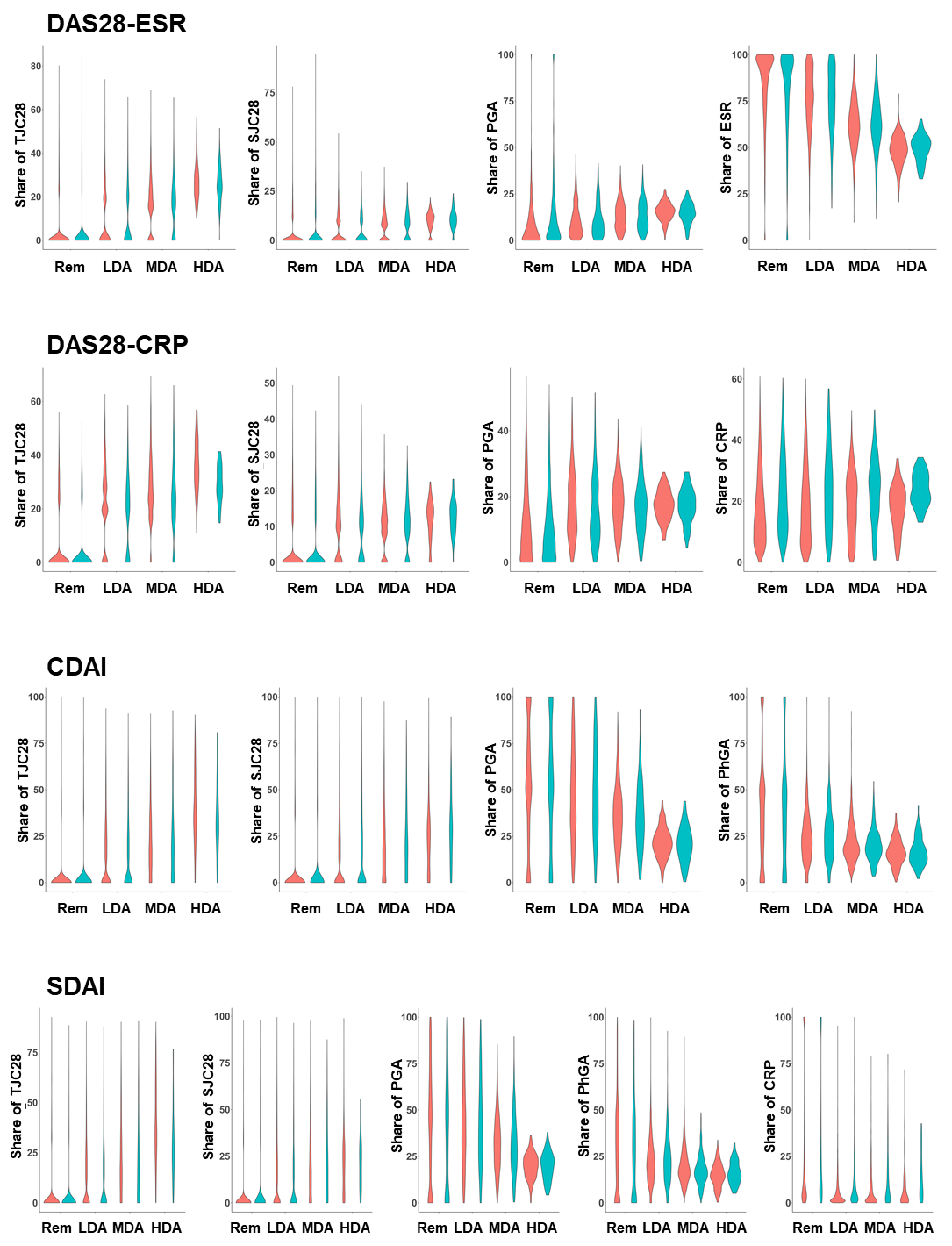


**Supplementary Figure 3.** Relationships between DAS28-ESR and age or joint findings before and after correction


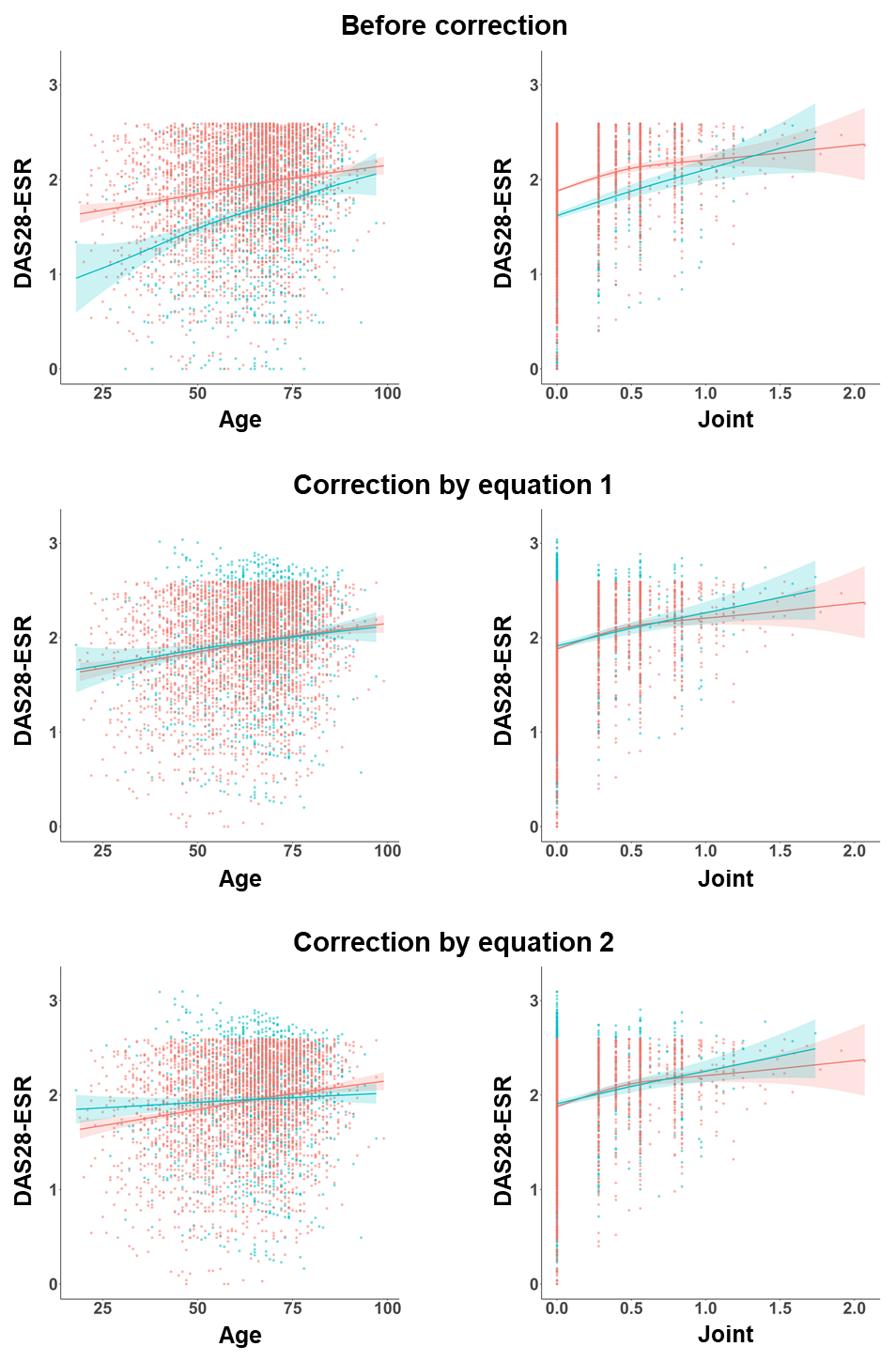


Supplementary Figure 1.

Legend:

Black line is drawn at the value of 0.000.

Blue, green, and red dashed lines are drawn on the values of 0.147, 0.330, and 0.474, respectively.

Points and bars indicate estimates and 95% CIs of Cliff’s delta, respectively.

Rem, remission; LDA, low disease activity; MDA, moderate disease activity; HDA, high disease activity.

Supplementary Figure 2.

Legend:

Red and blue violin plots depict distributions of the share of each component in females and males, respectively.

PGA, patient global assessment; PhGA, physician global assessment; Rem, remission; LDA, low disease activity; MDA, moderate disease activity; HDA, high disease activity.

Supplementary Figure 3.

Legend:

“Joint” represents 0.56 × √(TJC28) + 0.28 × √(SJC28).

Red and blue dots in scatter plots indicate values of females and males, respectively.

Curves with 95% confidence bands were fitted with generalized additive model by sex (red, female; blue, male).

**Supplementary Table 1.** Patient characteristics (DMARDs free)

**Supplementary Table 2.** Patient characteristics (csDMARDs)

**Supplementary Table 3.** Patient characteristics (TNFi)

**Supplementary Table 4.** Patient characteristics (IL-6i)

**Supplementary Table 5.** Patient characteristics (CTLA-4-Ig)

**Supplementary Table 6.** Patient characteristics (JAKi)

**Supplementary Table 7.** Cliff’s delta for sex difference in disease activity indices and their components by treatment type

**Supplementary Table 7.** Cliff’s delta for sex difference in disease activity indices and their components by treatment type (Continued)

**Supplementary Table 7.** Cliff’s delta for sex difference in disease activity indices and their components by treatment type (Continued)

**Supplementary Table 7.** Cliff’s delta for sex difference in disease activity indices and their components by treatment type (Continued)

**Supplementary Table 8.** PERMANOVA for sex difference in the share of components

**Supplementary Table 9.** Adjustment for patient-related factors with GLM and QR in available-case analysis

 **Supplementary Table 9.** Adjustment for patient-related factors with GLM and QR in available-case analysis (Continued)

**Supplementary Table 10.** Adjustment for patient-related factors with GLM and QR using stacked dataset imputed by chained equations

Supplementary Table 1-6.

Legend:

The values are n, n (%), mean (SD) or median (Q1-Q3). Number of artificial joints represents median, Q1-Q3 and range. SD, standard deviation; Q1, first quartile; Q3, third quartile; 95% CI, 95% confidence interval.

If categorical data contains missing data, the percentages are calculated with its denominator as the number subtracting the number of missing data from total number. If continuous data contains missing data, the representative values are the results of available-case analysis.

The value of difference in 25% trimmed mean (Δ25% trimmed mean) is calculated by subtracting the male value from the female value.

Supplementary Table 7.

Legend:

The values represent estimate (95% CI) of Cliff’s delta.

Positive values of Cliff’s delta indicate values of the indices or components are higher in females compared to males, whereas negative values indicate the opposite.

NA in estimates and/or 95% CIs shows that Cliff’s delta could not be calculated due to small sample size.

ALL means patients of all treatment type in available-case analysis.

PGA, patient global assessment; PhGA, physician global assessment; LDA, low disease activity; MDA, moderate disease activity; HDA, high disease activity.

Supplementary Table 8.

Legend:

Values represent R^2^ value (*p*-value).

Rem, remission; LDA, low disease activity; MDA, moderate disease activity; HDA, high disease activity.

Supplementary Table 9.

Legend:

Values represent estimate (95% CI) of partial regression coefficients for each variable.

The variable “Male” is dummy variable that female is used as reference.

The variables “Stage Ⅱ”, “Stage Ⅲ”, and “Stage Ⅳ” are dummy variables that Steinbrocker stage Ⅰ is used as reference.

The variable “NSAIDs use” and “Steroid use” are dummy variables that patients who do not use regularly NSAIDs and steroid are used as reference, respectively.

GLM, generalized linear model; QR, quantile regression; Rem, remission; LDA, low disease activity; MDA, moderate disease activity; HDA, high disease activity.

Supplementary Table 10.

Legend:

Values represent estimate of partial regression coefficients for each variable.

95% CIs are not shown because confidence intervals estimated by stacked imputed dataset would be invalid.

The variable “Male” is dummy variable that female is used as reference.

The variable “Stage Ⅱ”, “Stage Ⅲ”, “Stage Ⅳ” are dummy variables that Steinbrocker stage Ⅰ is used as reference.

The variable “NSAIDs use” and “Steroid use” are dummy variables that patients who do not use regularly NSAIDs and steroid are used as reference, respectively.

GLM, generalized linear model; QR, quantile regression; Rem, remission; LDA, low disease activity; MDA, moderate disease activity; HDA, high disease activity.

**URLs and/or references of R packages and function**

References

[1] Wickham H. ggplot2: Elegant Graphics for Data Analysis. New York, NY: Springer-Verlag 2016.

[2] Fox J, Weisberg S. An R companion to applied regression, Third Edition. Thousand Oaks, CA: Sage 2019.

[3] Peng RD. Simpleboot: simple bootstrap routines. R package version 1.1-7, 2019.

[4] Canty A, Ripley B. Boot: bootstrap R (S-Plus) functions. R package version 1.3-25, 2020.

[5] Davison AC, Hinkley DV. Bootstrap methods and their application. Cambridge, UK: Cambridge University Press 1997.

[6] Torchiano M. Effsize: efficient effect size computation. R package version 0.8.1, 2020.

[7] Oksanen J, Blanchet FG, Friendly M, et al. Vegan: community ecology package. R package version 2.5-7, 2020.

[8] Koenker R. Quantreg: quantile regression. R package version 5.85, 2021.

[9] van Buuren S, Groothuis-Oudshoorn K. mice: Multivariate imputation by chained equations in R. J Stat Software 2011;45(3):1–67.
